# Potential Molecular Mechanism of Upregulated Aryl Hydrocarbon Receptor Nuclear Translocator 2 in Nasopharyngeal Carcinoma

**DOI:** 10.1155/2022/9137282

**Published:** 2022-09-27

**Authors:** Si-Wei Huang, Gang Chen, Jian-Di Li, Li-Ting Qin, Zhi-Guang Huang, Su-Ning Huang, Wei Lu, Jiang-Hui Zeng, Bin-Yu Mo, Yi-Wu Dang, Zhu-Xin Wei, Jia-Yuan Luo

**Affiliations:** ^1^Department of Pathology, The First Affiliated Hospital of Guangxi Medical University, No. 6 Shuangyong Road, Nanning, Guangxi Zhuang Autonomous Region 530031, China; ^2^Department of Radiotherapy, Guangxi Medical University Cancer Hospital, No. 71 Hedi Rd, Nanning, Guangxi Zhuang Autonomous Region 530021, China; ^3^Department of Pathology, The Third Affiliated Hospital of Guangxi Medical University/Nanning Second People's Hospital, No. 13 Dancun Road, Nanning, Guangxi Zhuang Autonomous Region 530031, China; ^4^Department of Clinical Laboratory, The Third Affiliated Hospital of Guangxi Medical University/Nanning Second People's Hospital, No. 13 Dancun Road, Nanning, Guangxi Zhuang Autonomous Region 530031, China; ^5^Department of Otolaryngology, Liuzhou People's Hospital of Guangxi, No. 8 Wenchang Rd, Liuzhou, Guangxi Zhuang Autonomous Region 545006, China; ^6^Department of Radiotherapy, The First Affiliated Hospital of Guangxi Medical University, No. 6 Shuangyong Road, Nanning, Guangxi Zhuang Autonomous Region 530031, China

## Abstract

**Background:**

Currently, the benefits of nasopharyngeal carcinoma (NPC) therapy are limited, and it is necessary to further explore possible therapeutic targets. Aryl hydrocarbon receptor nuclear translocator 2 (ARNT2) has been extensively studied in other cancer species, but little has been explored in NPC. The aim of this study was to verify the expression level of ARNT2 and its underlying mechanism in NPC.

**Methods:**

Datasets containing ARNT2 mRNA expression levels were retrieved and collected from various databases to explore the expression status of ARNT2 in NPC. ARNT2-related coexpressed genes, differential expressed genes, and target genes were obtained for functional enrichment analysis. The potential target gene of ARNT2 and their regulatory relationship were studied through ChIP-seq data. CIBERSORTx was used to assess the immune infiltration of NPC, and the association with ARNT2 expression was calculated through correlation analysis.

**Results:**

ARNT2 was upregulated and possessed an excellent discriminatory capability in NPC samples. ARNT2 positively correlated target genes were clustered in pathways in cancer, while negatively correlated target genes were enriched in immune-related pathway. The ChIP-seq information of ARNT2 and histone showed that prostaglandin-endoperoxide synthase 2 (PTGS2) was a potential target gene of ARNT2. CIBERSORTx revealed the immunity status in NPC, and ARNT2 expression was correlated with infiltration of five immune cells.

**Conclusions:**

ARNT2 is overexpressed in NPC and may regulate PTGS2 to participate in the cancer process. ARNT2 serves as a key oncogenic target in NPC patients.

## 1. Introduction

Nasopharyngeal carcinoma (NPC) is an epithelial cancer that originates from nasopharyngeal mucosa. In 2020, there were about 130,000 new cases and 80,000 deaths of NPC worldwide [1]. However, the global geographic distribution of NPC shows a clear imbalance, and most of the cases are concentrated in East Asia and Southeast Asia and are closely related to previous infections with the Epstein-Barr virus [2, 3]. Although the mortality rate of NPC has been declining in recent years, about 10-20% of patients still relapse after receiving initial treatment, most of the targeted drugs are still in the basic experimental stage, and the precise mechanisms of action need to be further studied [4]; currently, the therapy for NPC mainly contains radiotherapy and chemotherapy, which brings a greater economic and health burden to patients [5]. Therefore, it is urgent to find the more key therapeutic target of NPC to improve the treatment.

Aryl hydrocarbon receptor nuclear translocator 2 (ARNT2) is a member of the basic helix-loop-helix period-ARNT-single-minded protein (bHLH/PAS) transcription factor family. ARNT2 is heterodimer with other bHLH/PAS members and directly transcribes target genes under various environmental and physiological stimuli [6]. Some studies have shown that ARNT2 plays a key role in tumor progression. For example, researchers suggested that ARNT2 may be a direct target of p53, which allows tumor angiogenesis [7]; Qin et al. found that ARNT2 can act through metabolism regulated by hypoxia-inducible factor-1 to affect the progression of breast cancer [8]. However, the previous research on the role of ARNT2 in different cancers was not consistent: ARNT2 was considered as an important tumorigenic factor in glioblastoma [9], and it significantly upregulated in PM2.5-induced lung cancer [10]. However, the upregulation of ARNT2 was associated with a better prognosis of patients, and its exogenous overexpression can inhibit the proliferation and aggressiveness of cancer cells in oral squamous cell carcinoma [11], liver cancer [12], and gastric cancer [13]. These may be related to the tissue specificity of ARNT2. Only one previous study pointed out the role of ARNT2 in NPC cisplatin resistance [14]. Nevertheless, the study lacked the verification of ARNT2 expression status in large sample size. The expression and potential mechanism of ARNT2 still need to be further explored.

In this article, we collected a large amount of ARNT2 expression data in NPC and nonnasopharyngeal carcinoma tissues or cell lines through public datasets. Through comprehensive analysis, we detected that ARNT2 was upregulated in NPC tissue and cells. Meanwhile, we further explored the possible mechanism of ARNT2 and its potential target genes in NPC. Subsequently, we confirmed the inflammatory factor prostaglandin-endoperoxide synthase 2 (PTGS2) as one of the potential target genes of ARNT2 by ChIP-seq data, and it was also supported by correlation calculations. Further, we utilized CIBERSORTx to reveal the immune landscape of NPCs and analyzed the association of ARNT2 with several immune cell fractions. Through the above research, we believe that ARNT2 is a key oncogenic target in NPC. The research design process of this paper is shown in supplementary figure 1.

## 2. Materials and Methods

### 2.1. Detecting ARNT2 mRNA Expression Level through Public Microarray Data

We searched and downloaded mRNA sequencing data from public databases such as The Cancer Genome Atlas (TCGA), Gene Expression Omnibus (GEO), ArrayExpress, Sequence Read Archive (SRA), and Oncomine. The search formula based on the MESH term was as follows: ((nasopharynx OR nasopharyngeal) AND (neoplasm OR cancer OR adenoma OR carcinoma OR tumor OR NPC OR neoplasia OR malignant OR malignancy)). Screening studies were based on the following criteria: (a) the specimen is from a human tissue or cell line, (b) can download the expression profile, and (c) can get the expression level of ARNT2. If the dataset was duplicated, only the latest version was kept. Some studies were excluded due to the following reasons: (a) samples from a treated patient or cell line, (b) normal control or cancer tissue sample size < 3, and (c) lack of ARNT2 data. The process of filtering the dataset is shown in supplementary figure 2. The included dataset was check carefully, and if any matrix was not standardized, log2 conversion was perform. In addition, the datasets were integrated into a larger matrix according to same platforms, and the limma voom package and sva package in Rv4.0.3 were used to eliminate batch effects between studies. Subsequently, the ARNT2 expression value was extracted and grouped according to the type of specimen. GraphPad Prism 8.0 was utilized to draw the box graphs and calculate the *t* test. The ROC curve was also drawn using GraphPad Prism 8.0, and the area under the curve (AUC) was calculated. AUC values less than 0.7, between 0.7 and 0.9, and greater than 0.9 represented the weak, moderate, and strong discriminatory capability of ARNT2 between NPC patients and control samples, respectively. We used STATA v15.0 to calculate the standardized mean difference (SMD) and compared the expression of ARNT2 mRNA in the NPC and control groups. The heterogeneity among the included studies was assessed by *I*^2^ statistics. The statistical significance is set as the *I*^2^ value is greater than 50%, and the *p* value is less than 0.05. In the case of significant heterogeneity, a random effects model was performed. Sensitivity analysis was used to explore potential sources of heterogeneity, and publication bias test was applied to assess the stability of SMD results. Simultaneously, the summary receiver operating characteristic (SROC) curve, the diagnostic specificity and sensitivity, and the Fagan graph were created to evaluate the ability of ARNT2 expression levels to identify cancer and noncancer samples [15–17].

### 2.2. Identification of Potential ARNT2-Related Dysregulated Target Genes

In the 9 datasets, we utilized the Pearson algorithm of R software to obtain ARNT2-related genes and selected ∣*r* | ≥0.3 and *p* < 0.05 as the criteria. The limma package was used to initially screen out the differential genes. The screening conditions for these genes were |logFoldChange| > 1 and adjusted *p* < 0.05. Subsequently, the SMD of each differential gene was calculated, and those differential genes whose 95% confidence interval (CI) crossed 0 were eliminated. Meanwhile, we searched the ChIP-seq data of ARNT2 in Cistrome DB (http://cistrome.org/db/) [18] and got putative target genes of ARNT2, among which genes with score ≤ 0 were excluded. ARNT2 positively related genes with the number of repetitions not less than 2 times were intersected with the upregulated differential genes and the target genes in NPC. Similarly, ARNT2 negatively related genes, downregulated genes, and target genes were intersected. Thus, we got two gene sets.

### 2.3. Potential Molecular Mechanism of ARNT2 in NPC

We used the two sets of crossover genes obtained above to perform Gene Ontology (GO) and Kyoto Encyclopedia of Genes and Genomes (KEGG) enrichment analyses through DAVID Bioinformatics Resources 6.8 (https://david.ncifcrf.gov/); the top ten pathways with the most significant enrichment were selected and visualized using the ggplot package of the R software environment. STRING (https://cn.string-db.org/) was used to construct a protein-to-protein interaction (PPI) network based on the first three KEGG pathway genes [19–21].

### 2.4. Acquisition of the Target Gene PTGS2

We integrated the foregoing putative target genes of ARNT2 in ChIP-seq data. In addition, histone H3K27ac and H3K4ME3 information of NPC cell line C666-1 were also collected (GSM2523126 and GSM2523127); they could be used to characterize the transcriptional activation status of genes in order to find ARNT2 target genes more accurately. In order to visually display the results, we applied Integrative Genomics Viewer (IGV) to analyze and display the ChIP-seq information of ARNT2 and the target gene. JASPAR (http://jaspar.genereg.net/) is a database that collects transcription factor binding profiles [22], through which we obtained the motif information of ARNT2. In order to obtain the expression of PTGS2 and its correlation with ARNT2, we extracted the expression value of PTGS2 to calculate SMD and draw SROC curve. GraphPad Prism 8.0 was used to calculate the correlation coefficient between PTGS2 and ARNT2 and draw a scatter plot. Since the correlation coefficient *r* and the standard error (se) cannot be directly used for merging, we converted the *r* value to Fisher's *Z*, then calculated the se value of *Z*, and performed the SMD calculation of *r* through the *Z* and se values. The final result was transformed into *r*.

### 2.5. Assessment of Correlation between ARNT2 and Immune Infiltration

We used the aforementioned integrated matrix GPL1154 for the assessment of the immune infiltration landscape in NPC because it has the largest sample size. We uploaded the gene expression file to CIBERSORTx (https://cibersortx.stanford.edu/), a deconvolution algorithm based on gene expression data that can be characterized using a “feature matrix” consisting of specific gene expression values of immune cells. We used 1000 permutation parameters and the default LM22 signature matrix file for calculation. Those samples with *p* > 0.05 were eliminated. GraphPad Prism 8.0 was used to visualize the differences between each immune cell in NPC versus controls and in ARNT2 high versus low expression groups. The infiltration fraction of immune cells in cancer samples and the corresponding ARNT2 expression were extracted for correlation analysis.

## 3. Results

### 3.1. Upregulation of ARNT2 mRNA in NPC

**Figure 1:**
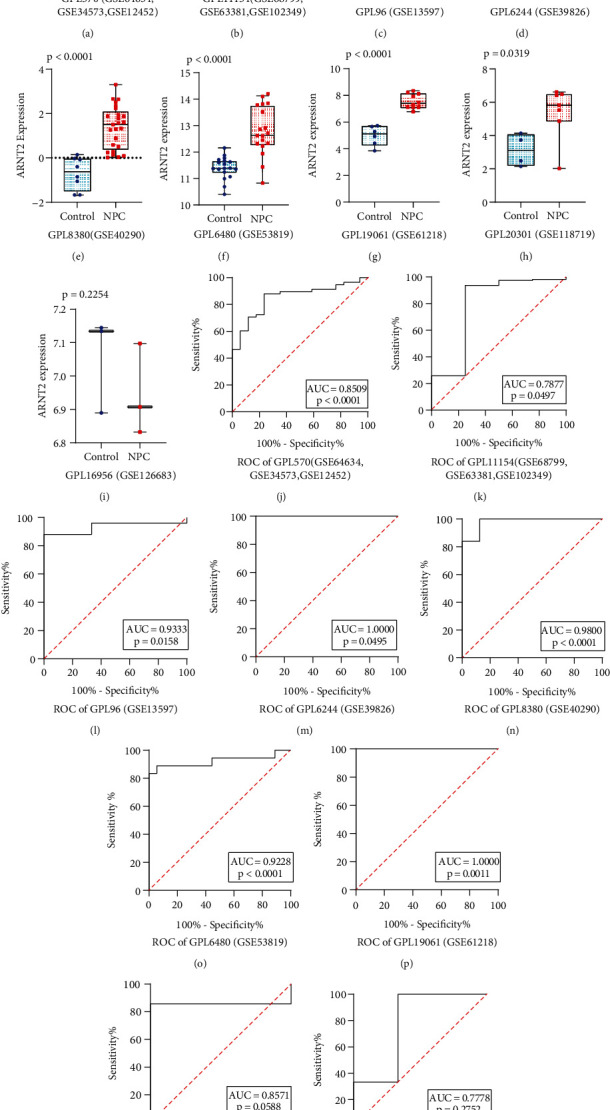
ARNT2 expression levels in NPC based on multiple cohorts. (a~i) Different expression levels of ARNT2 between NPC and nontumor nasopharynx samples. (j~r) Operating characteristic curves of ARNT2 mRNA expression for the differentiation of nasopharynx carcinoma from nontumor tissues.

**Figure 2:**
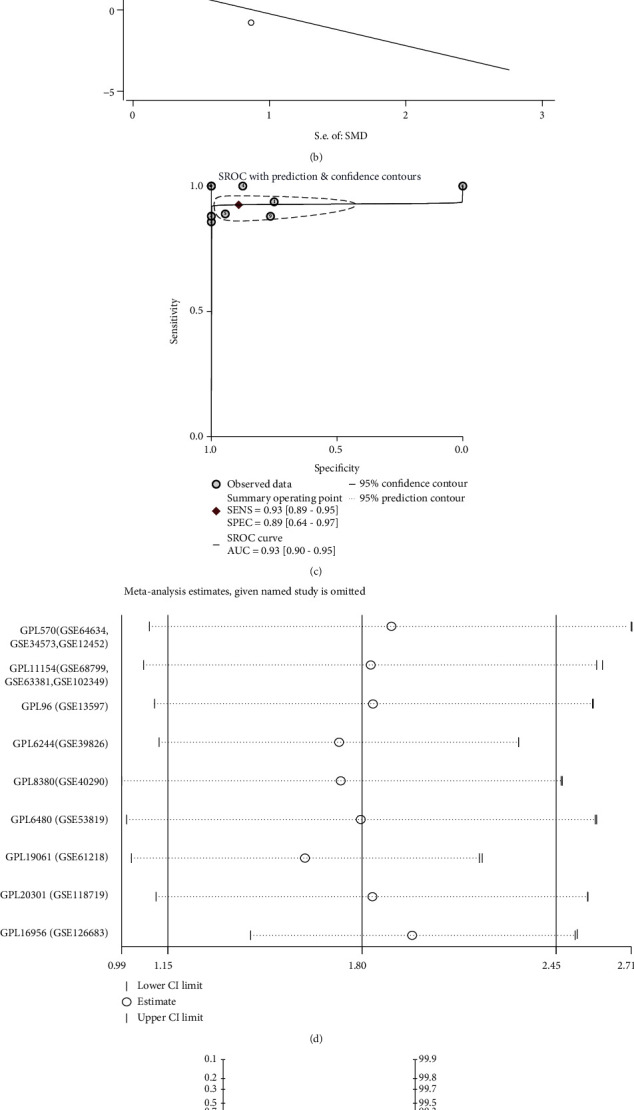
Comprehensive ARNT2 expression level in NPC tissues based on multiple gene chips. (a) Forest plot for assessing ARNT2 expression between NPC tissues and nontumor tissues. (b) Analysis of the publication bias detection in the comprehensive analysis evaluating the expression pattern of ARNT2 in NPC. (c) Summary receiver operating characteristic curve of the distinguishing capability of ARNT2 for cancer from noncancerous samples. (d) Sensitivity analysis of standard mean deviation (random effects model). (e) Fagan's nomogram.

A total of 13 gene chips containing ARNT2 mRNA expression patterns were included. Among them, GSE68799, GSE63381, and GSE102349 were combined into an array and named GPL11154. Similarly, GSE64634, GSE34573, and GSE12452 were combined and named GPL570. The rest of the datasets were not merged. After the above processing, nine datasets were obtained, including a total of 308 NPC samples and 66 noncancer controls (Table 1). We extracted the expression value of ARNT2 in each dataset and created a box chart with scatter points (Figures 1(a)–1(i)). The results showed that in eight of the nine datasets, the ARNT2 mRNA expression level of NPC was higher than that of the noncancer groups. Due to the significant heterogeneity (*I*^2^ = 63.4%, *p* = 0.005), a random effects model was used. The SMD value of 1.80 (95% CI: 1.15–2.45) indicates that the expression of ARNT2 in NPC tissues is significantly higher than that in noncancerous tissues (Figure 2(a)). Begg's test manifested that no publication bias existed (Figure 2(b)). Sensitivity analysis showed that the included studies cannot explain the source of heterogeneity (Figure 2(d)). The ROC curves of nine datasets indicated the ability of ARNT2 to distinguish between NPC and control samples, and five of them showed the strong ability of ARNT2 to separate the NPC group from noncancer control (Figures 1(j)–1(r)). The area under the sROC curve was 0.93 (95% CI: 0.90–0.95, Figure 2(c)), which revealed the remarkable ability of ARNT2 to distinguish NPC individuals from noncancer individuals. Among them, the diagnostic sensitivity was 0.93 (95% CI: 0.89-0.95), and the specificity was 0.89 (95% CI: 0.64-0.97). In Fagan chart, the pretest probability was 20%, the posttest probability of using ARNT2 to detect NPC positive results was 68%, and the probability of negative results was 2% (Figure 2(e)), indicating that ARNT2 could be applied as a significant marker for NPC screening.

### 3.2. Acquisition of ARNT2 Potential Target Genes

The correlation between all genes and ARNT2 in 9 datasets was calculated, and finally, 3,527 positively related genes and 3334 negatively related genes that met the criteria which was described in the methods were obtained. Meanwhile, we got 1904 high-expressed differential genes and 1655 low-expressed differential genes. Through the Cistrome DB database, we obtain the ARNT2 ChIP-seq sample GSM1239447, in which there are 7899 putative target genes with a score > 0. After the intersection process, 558 up DEGs-positive CEGs-target genes and 515 down DEGs-negative CEGs-target genes were selected.

### 3.3. Analysis of the Potential Mechanism of ARNT2 Target Genes in NPC

**Figure 3:**
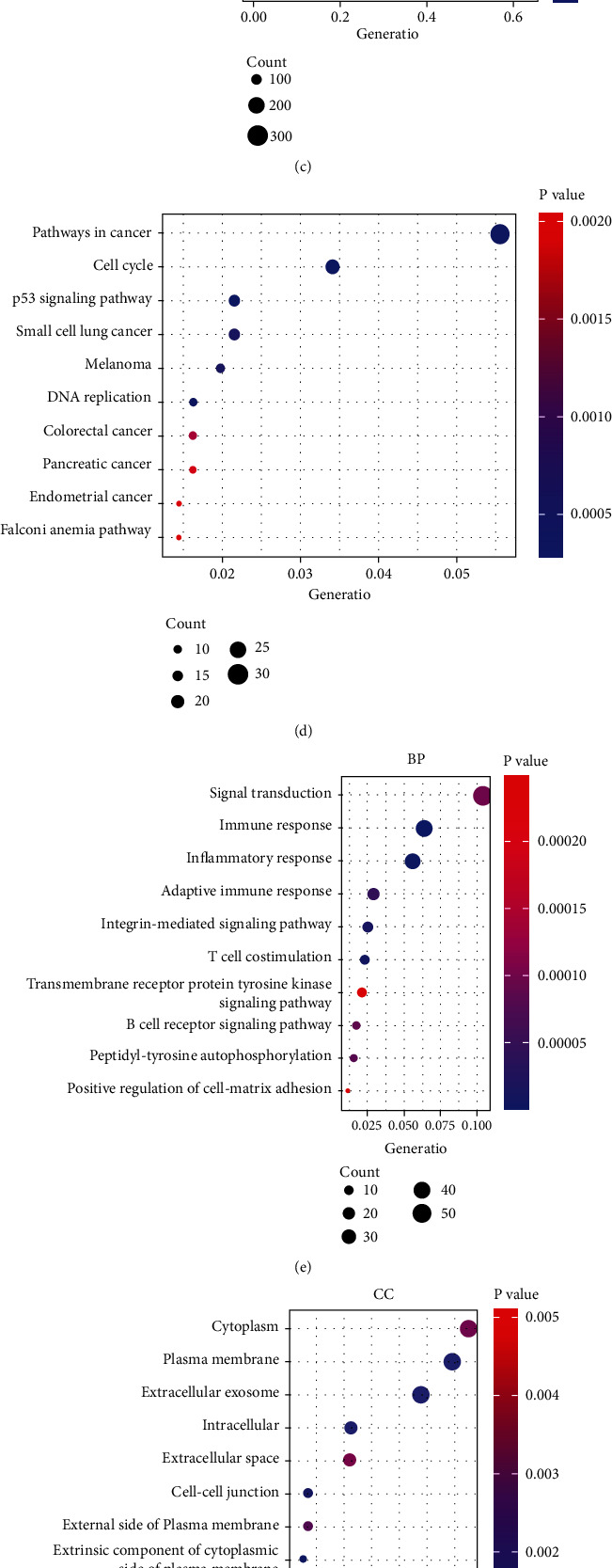
Potential gene annotations and prospective pathways of ARNT2-related target genes in NPC. (a~c) Gene Ontology based on intersection of upregulated DEGs, ARNT2 positively correlated CEGs, and target genes. (d) Kyoto Encyclopedia of Genes and Genomes based on intersection of upregulated DEGs, ARNT2 positively correlated CEGs, and target genes. (e~g) Gene Ontology based on intersection of downregulated DEGs, ARNT2 negatively correlated CEGs, and target genes. (h) Kyoto Encyclopedia of Genes and Genomes based on intersection of downregulated DEGs, ARNT2 negatively correlated CEGs, and target genes. Note: count refers to the number of genes enriched in the pathway.

We performed KEGG and GO pathway analyses through DAVID based on the two sets of genes obtained above. According to the enrichment analysis of 558 up DEGs-positive CEGs-target genes, GO analysis showed that they were significantly enriched in signal transduction (Biological Process, BP), nucleus (Cellular Component, CC), and protein binding (Molecular Function, MF; Figures 3(a)–3(c)); the KEGG enrichment analysis showed that the most abundant genes were enriched in the pathways in cancer (Figure 3(d)), suggesting that ARNT2 and its positively related and upregulated target genes may be involved in the occurrence and development of cancer, while 515 down DEGs-negative CEGs-target genes showed a preference for immune-related pathways in the enrichment analysis: such as immune response, adaptive immune response, and T cell costimulation in BP and NK cell-mediated cytotoxicity and B cell costimulation in KEGG (Figures 3(e)–3(h)). The two sets of genes enriched in the first three pathways of KEGG were integrated to construct a PPI network to unfold the interaction among proteins (supplementary figure 3A and 3B).

### 3.4. The Targeted Regulation of ARNT2 on PTGS2

**Figure 4:**
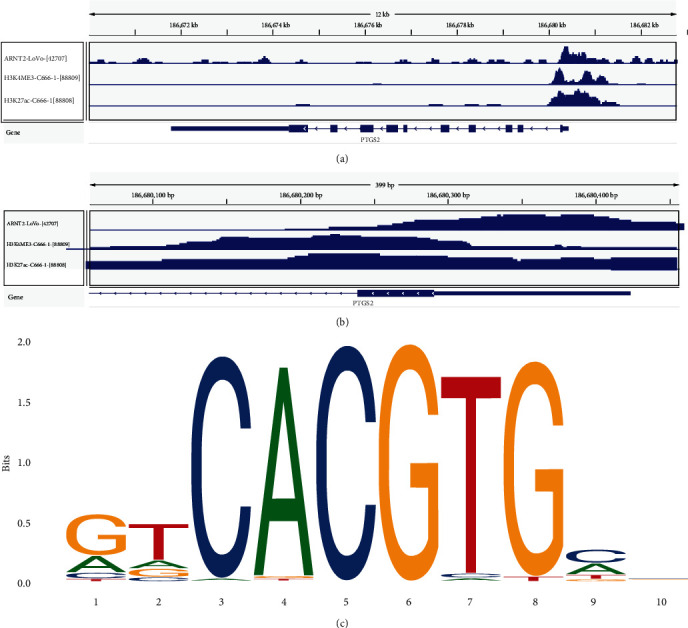
ARNT2 ChIP-seq information. (a) ChIP-seq for the full length of PTGS2 transcription. (b) A magnified view of the peak region. (c) Predictive motif of ARNT2.

**Figure 5:**
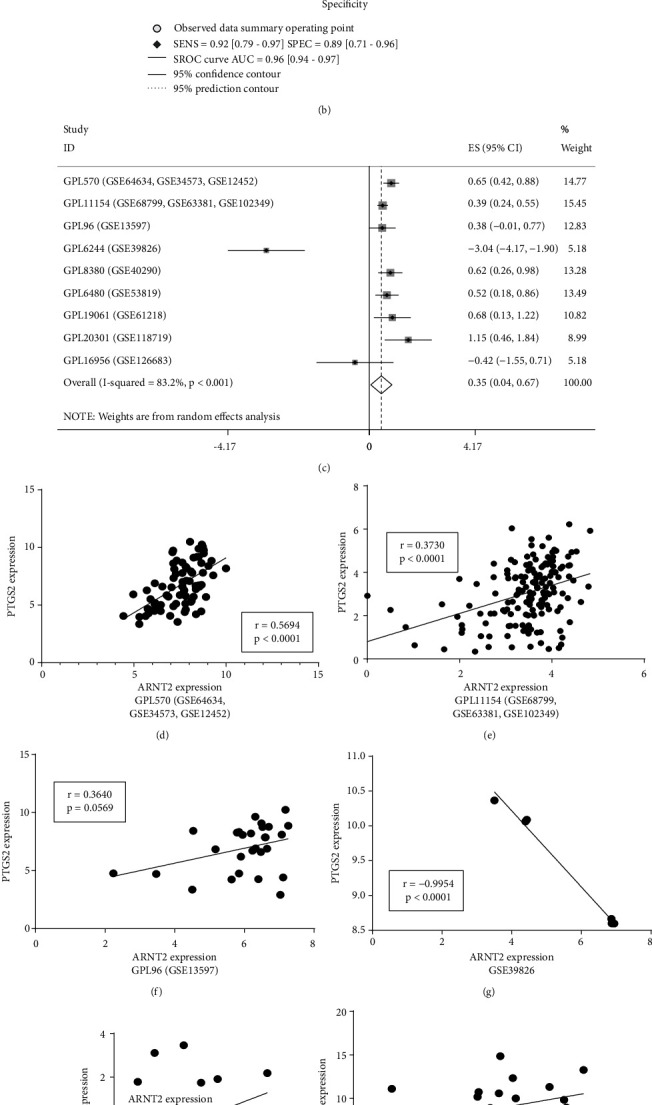
Comprehensive PTGS2 expression level and correlation analysis of ARNT2 and PTGS2 expression in NPC. (a) Forest plot for evaluating PTGS2 expression between NPC specimens and nontumor specimens. (b) Summary receiver operating characteristic curve of the distinguishing capability of PTGS2 for cancer from noncancerous tissues. (c) Forest plot for assessing correlation of ARNT2 and PTGS2 expression level. (d~l) A correlation analysis of ARNT2 and PTGS2 expression levels based on 9 datasets.

The ChIP-seq information about ARNT2 and PTGS2 is shown in Figures 4(a) and 4(b). The specific peaks in the promoter region of PTGS2 could be observed in three samples, whereas no distinct fluctuation existed in the middle and downstream of coding region.  Figure 4(c) displays the predicted motif diagram of ARNT2. We calculated the SMD of PTGS2 as 1.52 (95% CI: 0.95-2.10, Figure 5(a)). Simultaneously, SROC curve was also drawn, where AUC was 0.96 (95% CI: 0.94-0.97, Figure 5(b)). Both parameters reflected the fact that PTGS2 mRNA expression was upregulated in NPC tissues. In addition, we calculated the correlation coefficient *r* between ARNT2 and PTGS2 based on nine datasets and plotted a scatter plot (Figures 5(d)–5(l)). The results indicated that there was a positive correlation between ARNT2 and PTGS2 in seven datasets. Then, we integrate and calculate the obtained *r* and gained the pooled estimate of *r* to be 0.34 (95% CI: 0.04-0.58, Figure 5(c)), which manifested that the correlation between ARNT2 and PTGS2 was meaningful.

### 3.5. Immune Landscape in NPC and Its Association with ARNT2

**Figure 6:**
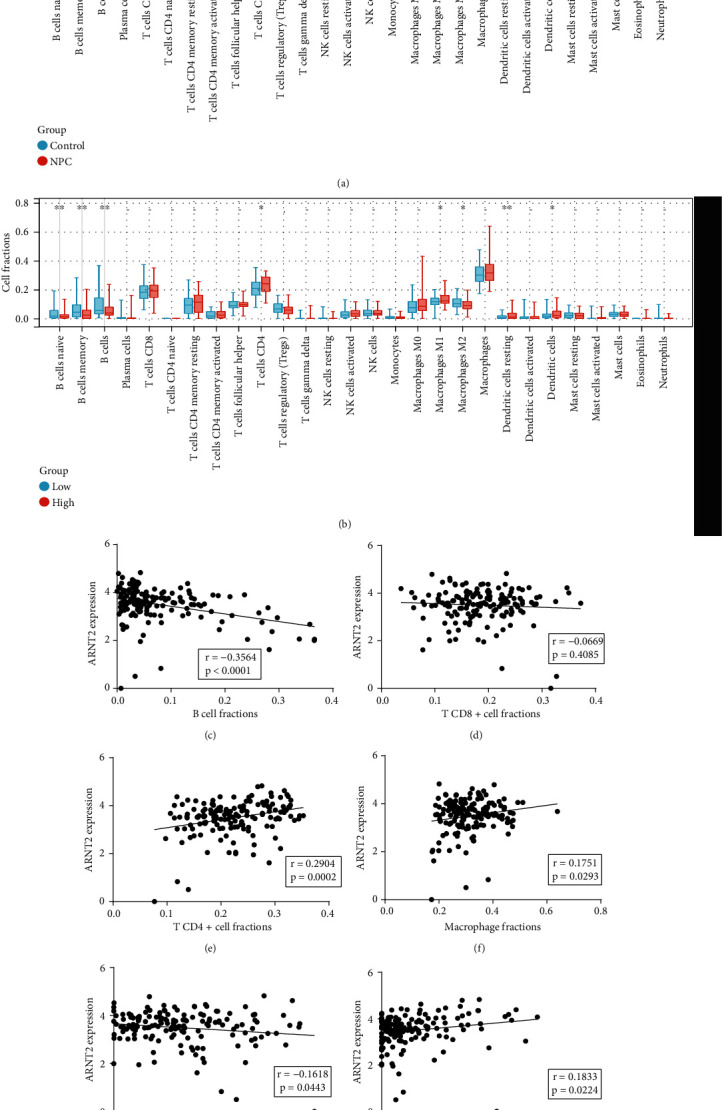
Analysis of immune infiltration and the correlation with ARNT2. (a) Comparison of various immune cell infiltration between the NPC and control groups. (b) Different immune-infiltrating landscape of low and high ARNT2 expression group in NPC samples. (c~h) Linear correlation between ARNT2 and six types of immune cell infiltration fraction.

The immune landscape of GPL11154 was analyzed by CIBERSORTx.  Figure 6(a) shows the difference in immune infiltration between NPC and noncancerous samples, compared with normal nasopharyngeal tissue; there was less B cell infiltration in NPC tissue, while CD4^+^ T cells, macrophages, mast cells, and dendritic cell infiltration was significantly increased. The NPC samples were divided into ARNT2 high and low expression group, and it was found that the samples with high ARNT2 expression had a lower proportion of B cells, and a higher proportion of CD4^+^ T cells and dendritic cells infiltrated (Figure 6(b)). The correlation between six major immune cells and ARNT2 was further analyzed, and the results showed that ARNT2 expression was positively correlated with CD4^+^ T cells, macrophages, and dendritic cells but negatively related with B cells and mast cells (Figures 6(c)–6(h)).

## 4. Discussion

In this study, we integrated 13 NPC datasets, including 308 NPC samples and 66 noncancer controls from four countries, which have the characteristics of multiple samples and centers; we applied comprehensive analysis to obtain the conclusion that ARNT2 mRNA was upregulated in NPC; then, we further explored the potential molecular mechanism of ARNT2: analyze the pathways where dysregulated target genes related to ARNT2 and in NPC were enriched. Finally, we found the overexpressed target genes that were positively correlated with ARNT2 enriched in pathways in cancers, cell cycle, and p53 signaling pathway markedly. Interestingly, in the enrichment analysis of low-expressed genes negatively correlated with ARNT2, it was observed that these genes were involved in immune-related functions, including the immune responses related to B cells, T cells, and NK cells. Combined with ChIP-seq and correlation analysis, we discovered a target gene of ARNT2, PTGS2, or cyclooxygenase-2. The immune infiltration analysis tool CIBERSORTx revealed the immune landscape in NPC, as well as the potential immune regulated function of ARNT2. We speculate that the upregulation of ARNT2 will promote the expression of PTGS2 and other key target genes, thereby promoting the progress of NPC.

As a member of the bHLH/PAS transcription factor family, ARNT2 regulates the transcription of its target genes, including hypoxia-inducible factor 1*α* (HIF-1*α*), which can combine with ARNT2 to form a complex and initiate hypoxia/nutrient deprivation-induced vascular endothelial growth factor expression, thereby promoting tumor angiogenesis [9, 13]. Similar to our study, Bogeas et al. suggested that, compared with noncancer cells, the expression of ARNT2 in glioblastoma cells was upregulated [9]; another study found that ARNT2 was overexpressed in PM2.5-induced lung cancer [10]. Interestingly, Li et al. found that ARNT2 was downregulated in the tissues and serum of NPC cisplatin-resistant patients [14]. We consider the reason is that ARNT2 may be a sensitive gene for cisplatin therapy. Our research detected the overexpression of ARNT2 in NPC, demonstrated the potential signaling pathways related to ARNT2 in NPC, and obtained more NPC biomarkers, which is helpful for understanding the underlying mechanism of ARNT2 in NPC.

After studying the ChIP-seq sample of ARNT2, PTGS2, also known as cyclooxygenase 2, attracted our attention: a strong signal was shown at PTGS2 transcription start site. Moreover, H3K27ac and H3K4ME3 assembled at the PTGS2 transcription initiation site in NPC cell line samples, and the histones usually mark the activation state of transcription [23, 24]. Correlation linear analysis also supports the positive regulation of ARNT2-PTGS2 axis. As a classic inflammatory factor, PTGS2 has been found to play pleiotropic and multifaceted roles in the occurrence or promotion of various cancers and the resistance of cancer cells to radiotherapy and chemotherapy. PTGS2 can induce an immunosuppressive state in the tumor environment by inducing the recruitment of immune cells into tumor tissues, thereby guiding the immunophenotype and favoring the activation of cancer cells [25]. Previous studies have found that PTGS2 could mediate the interaction between cancer cells and myeloid-derived suppressor cells to promote nasopharyngeal carcinoma metastasis [26]; PTGS2 overexpression was also associated with poor prognosis in NPC patients and led to chemotherapy resistance production [27]. The above findings all indicate that PTGS2 is an important tumor-promoting molecule and plays a key role in tumor immune regulation.

We applied CIBERSORTx to uncover the difference in immune infiltration between NPC and normal tissues, as well as the association with ARNT2: ARNT2 expression was positively correlated with CD4^+^ T cells, macrophages, and dendritic cells, while it exhibited negative correlation with B cells and mast cells. Previous studies have shown that B cells were often significantly reduced in the tumor microenvironment compared to T cells [28] and that B cells often play an active role in anti-NPC immunity [29]. Macrophages and dendritic cells were reported to be the most abundant myeloid lineage subtypes in the NPC microenvironment [29], and both were shown to infiltrate more in NPCs than controls in this study. Macrophages are involved in a variety of functions, from organogenesis and tissue homeostasis to recognition and destruction of invading pathogens. In cancer, tumor-associated macrophages can promote tumor progression by enhancing cancer cell migration and invasiveness, stimulating angiogenesis, and suppressing antitumor immunity. There is increasing evidence that these diverse functions can be regulated by molecules in the tumor microenvironment [30]. Dendritic cells, as classical antigen-presenting cells, activate naive T cells and initiate key programs of antitumor immune responses. Dendritic cells, however, also need to undergo a maturation process to facilitate T cell priming; in the absence of activation, dendritic cells promote tolerance and induce immunomodulatory responses [31]. In this study, immunological analysis found that the infiltration ratio of resting dendritic cells in NPC was higher than that of activated dendritic cells, and the former was also higher than that in the control group, which indicated a potential immunosuppressive state in NPC. In conclusion, we believe that ARNT2 regulates and activates the transcription of PTGS2, and the overexpression of the two may influence immune microenvironment to promote occurrence and development of NPC.

Our research still has some limitations. First, due to the lack of prognostic information for NPC patients, no survival analysis study was conducted. Second, ARNT2 was detected only at the level of mRNA, and its protein expression was not verified. Third, the calculation of SMD suggested that the included studies had high heterogeneity, but sensitivity analysis could not explain the source of heterogeneity; this may be because the studies we included are from different countries. Finally, the more precise molecular mechanism of ARNT2 in NPC still needs further exploration.

## 5. Conclusion

ARNT2 is upregulated in NPC, and it may promote progress of NPC with its positively correlated CEGs, upregulated DEGs, and target genes cross genes; among them, PTGS2 may be a key oncogenic target gene under the action of ARNT2. Next, we will conduct a series of in vitro and in vivo experiments to examine it.

## Figures and Tables

**Table 1:** Characteristics of high-throughput datasets included in the study.

Study	Country	platform	Cancer group	Normal control	Mean1 ± SD1	Mean0 ± SD0
GSE64634, GSE34573, GSE12452	China, UK, USA	GPL570	58	17	7.83 ± 1.02	6.41 ± 0.91
GSE68799, GSE63381, GSE102349	China, Singapore, USA	GPL11154	159	4	3.50 ± 0.72	2.24 ± 1.25
GSE13597	UK	GPL96	25	3	6.18 ± 1.03	4.54 ± 1.08
GSE39826	UK	GPL6244	3	3	6.89 ± 0.04	4.11 ± 0.53
GSE40290	China	GPL8380	25	8	1.38 ± 0.96	−0.70 ± 0.72
GSE53819	China	GPL6480	18	18	12.79 ± 0.92	11.40 ± 0.42
GSE61218	China	GPL19061	10	6	7.56 ± 0.56	4.98 ± 0.74
GSE118719	USA	GPL20301	7	4	5.40 ± 1.61	3.12 ± 0.96
GSE126683	China	GPL16956	3	3	6.95 ± 0.14	7.06 ± 0.14

Note: Mean1 ± SD1: nasopharyngeal carcinoma tissues; Mean0 ± SD0: nontumor tissues. SD: standard deviation.

## Data Availability

The datasets analyzed during the current study are available in the Gene Expression Omnibus (https://www.ncbi.nlm.nih.gov/geo/) and Cistrome Data Browser (http://cistrome.org/db/#/).

## Supplementary Materials

Supplementary Materials Supplementary Figure 1: flow chart of the study design. Supplementary Figure 2: inclusion and exclusion of datasets. Supplementary Figure 3: PPI network of ARNT2-related target genes in NPC. (A) PPI network based on the genes of the first three KEGG pathways of intersection of upregulated DEGs, ARNT2 positively correlated CEGs, and target genes. (B) PPI network based on the genes of the first three KEGG pathways of intersection of downregulated DEGs, ARNT2 negatively correlated CEGs, and target genes.

## References

[1] Sung H., Ferlay J., Siegel R. L. (2021). Global cancer statistics 2020: GLOBOCAN estimates of incidence and mortality worldwide for 36 cancers in 185 countries. *CA: a Cancer Journal for Clinicians*.

[2] Chen Y. P., Chan A. T., Le QT B. P., Sun Y., Ma J. (2019). Nasopharyngeal carcinoma. *The Lancet*.

[3] Richardo T., Prattapong P., Ngernsombat C. (2020). Epstein-Barr virus mediated signaling in nasopharyngeal carcinoma carcinogenesis. *Cancers*.

[4] Kang Y., He W., Ren C. (2020). Advances in targeted therapy mainly based on signal pathways for nasopharyngeal carcinoma. *Signal Transduction and Targeted Therapy*.

[5] Svajdova M., Sicak M., Dubinsky P., Slavik M., Slampa P., Kazda T. (2020). Recurrent nasopharyngeal cancer: critical review of local treatment options including recommendations during the COVID-19 pandemic. *Cancers*.

[6] Rahim T., Becquart P., Baeva M. E., Quandt J. (2018). Expression of the neuroprotective protein aryl hydrocarbon receptor nuclear translocator 2 correlates with neuronal stress and disability in models of multiple sclerosis. *Journal of Neuroinflammation*.

[7] Garritano S., Inga A., Gemignani F., Landi S. (2013). More targets, more pathways and more clues for mutant p53. *Oncogenesis*.

[8] Qin X. Y., Wei F., Yoshinaga J., Yonemoto J., Tanokura M., Sone H. (2011). siRNA-mediated knockdown of aryl hydrocarbon receptor nuclear translocator 2 affects hypoxia-inducible factor-1 regulatory signaling and metabolism in human breast cancer cells. *FEBS Letters*.

[9] Bogeas A., Morvan-Dubois G., El-Habr E. A. (2018). Changes in chromatin state reveal ARNT2 at a node of a tumorigenic transcription factor signature driving glioblastoma cell aggressiveness. *Acta Neuropathologica*.

[10] Chen Z., Ji N., Wang Z. (2018). Fine particulate matter (PM2.5) promoted the invasion of lung cancer cells via an ARNT2/PP2A/STAT3/MMP2 pathway. *Journal of Biomedical Nanotechnology*.

[11] Kimura Y., Kasamatsu A., Nakashima D. (2016). ARNT2 regulates tumoral growth in oral squamous cell carcinoma. *Journal of Cancer*.

[12] Li W., Liang Y., Yang B., Sun H., Wu W. (2015). Downregulation of ARNT2 promotes tumor growth and predicts poor prognosis in human hepatocellular carcinoma. *Journal of Gastroenterology and Hepatology*.

[13] Jia Y., Hao S., Jin G. (2019). Overexpression of ARNT2 is associated with decreased cell proliferation and better prognosis in gastric cancer. *Molecular and Cellular Biochemistry*.

[14] Li J., Hu C., Chao H. (2021). Exosomal transfer of miR-106a-5p contributes to cisplatin resistance and tumorigenesis in nasopharyngeal carcinoma. *Journal of Cellular and Molecular Medicine*.

[15] Liu A. G., Zhong J. C., Chen G. (2020). Upregulated expression of SAC3D1 is associated with progression in gastric cancer. *International Journal of Oncology*.

[16] Peng W., Li J. D., Zeng J. J. (2020). Clinical value and potential mechanisms of COL8A1 upregulation in breast cancer: a comprehensive analysis. *Cancer Cell International*.

[17] Zheng H. P., Huang Z. G., He R. Q. (2019). Integrated assessment of CDK1 upregulation in thyroid cancer. *American Journal of Translational Research*.

[18] Zheng R., Wan C., Mei S. (2019). Cistrome Data Browser: expanded datasets and new tools for gene regulatory analysis. *Nucleic Acids Research*.

[19] Gao R. Z., Que Q., Lin P. (2019). Clinical roles of miR-136-5p and its target metadherin in thyroid carcinoma. *American Journal of Translational Research*.

[20] Sun Y., Li S. H., Cheng J. W. (2020). Downregulation of miRNA-205 expression and biological mechanism in prostate cancer tumorigenesis and bone metastasis. *BioMed Research International*.

[21] Liu A. G., Pang Y. Y., Chen G. (2020). Downregulation of miR-199a-3p in hepatocellular carcinoma and its relevant molecular mechanism via GEO, TCGA database and in silico analyses. *Technology in Cancer Research & Treatment*.

[22] Fornes O., Castro-Mondragon J. A., Khan A. (2019). JASPAR 2020: update of the open-access database of transcription factor binding profiles. *Nucleic Acids Research*.

[23] Tafessu A., Banaszynski L. A. (2020). Establishment and function of chromatin modification at enhancers. *Open Biology*.

[24] Leong M. M. L., Lung M. L. (2021). The impact of Epstein-Barr virus infection on epigenetic regulation of host cell gene expression in epithelial and lymphocytic malignancies. *Oncologia*.

[25] Hashemi Goradel N., Najafi M., Salehi E., Farhood B., Mortezaee K. (2019). Cyclooxygenase-2 in cancer: a review. *Journal of Cellular Physiology*.

[26] Li Z. L., Ye S. B., OuYang L. Y. (2015). COX-2 promotes metastasis in nasopharyngeal carcinoma by mediating interactions between cancer cells and myeloid-derived suppressor cells. *Oncoimmunology*.

[27] Shi C., Guan Y., Zeng L. (2018). High COX-2 expression contributes to a poor prognosis through the inhibition of chemotherapy-induced senescence in nasopharyngeal carcinoma. *International Journal of Oncology*.

[28] Sharonov G. V., Serebrovskaya E. O., Yuzhakova D. V., Britanova O. V., Chudakov D. M. (2020). B cells, plasma cells and antibody repertoires in the tumour microenvironment. *Nature Reviews. Immunology*.

[29] Gong L., Kwong D. L., Dai W. (2021). Comprehensive single-cell sequencing reveals the stromal dynamics and tumor-specific characteristics in the microenvironment of nasopharyngeal carcinoma. *Nature Communications*.

[30] Pan Y., Yu Y., Wang X., Zhang T. (2020). Tumor-associated macrophages in tumor immunity. *Frontiers in Immunology*.

[31] Lee Y. S., Radford K. J. (2019). The role of dendritic cells in cancer. *International Review of Cell and Molecular Biology*.

