# Supplementary material for: Potential Molecular Mechanism of Upregulated Aryl Hydrocarbon Receptor Nuclear Translocator 2 in Nasopharyngeal Carcinoma
Source: Comput Math Methods Med. 2022 Sep 27;2022:9137282. doi: 10.1155/2022/9137282 (PMC9532129; doi:10.1155/2022/9137282)
Supplement: Supplementary Materials — Supplementary Figure 1: flow chart of the study design. Supplementary Figure 2: inclusion and exclusion of datasets. Supplementary Figure 3: PPI network of ARNT2-related target genes in NPC. (A) PPI network based on the genes of the first three KEGG pathways of intersection of upregulated DEGs, ARNT2 positively correlated CEGs, and target genes. (B) PPI network based on the genes of the first three KEGG pathways of intersection of downregulated DEGs, ARNT2 negatively correlated CEGs, and target genes. [file 9137282.f1.pdf]

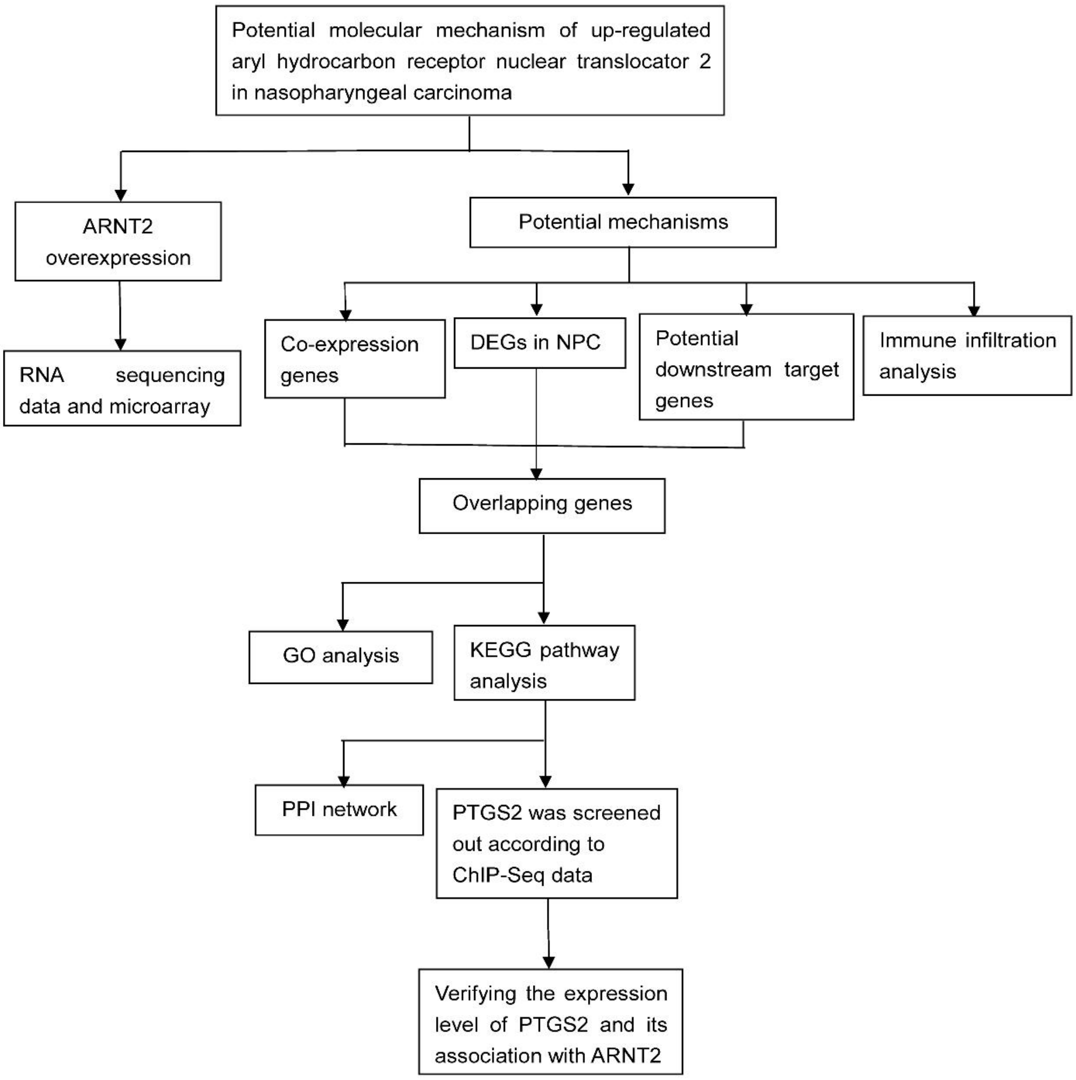

Supplementary Figure 1: Flow chart of the study design.

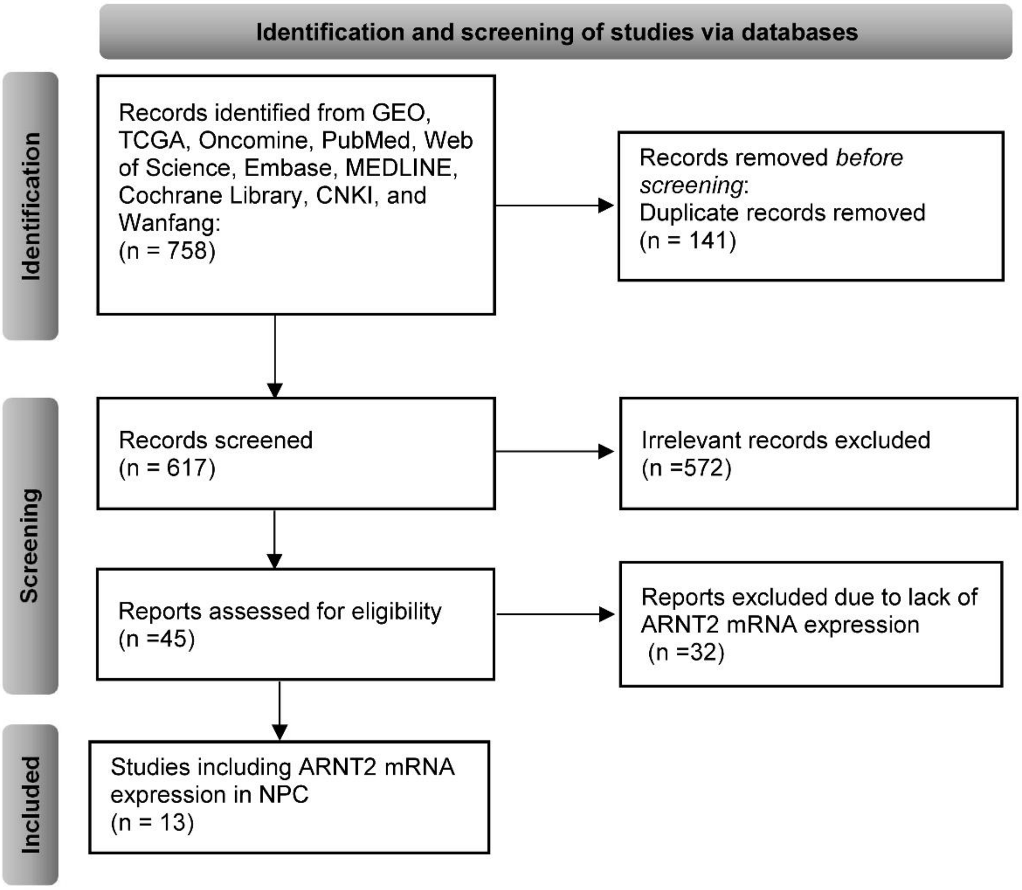

Supplementary Figure 2： Inclusion and exclusion of datasets.

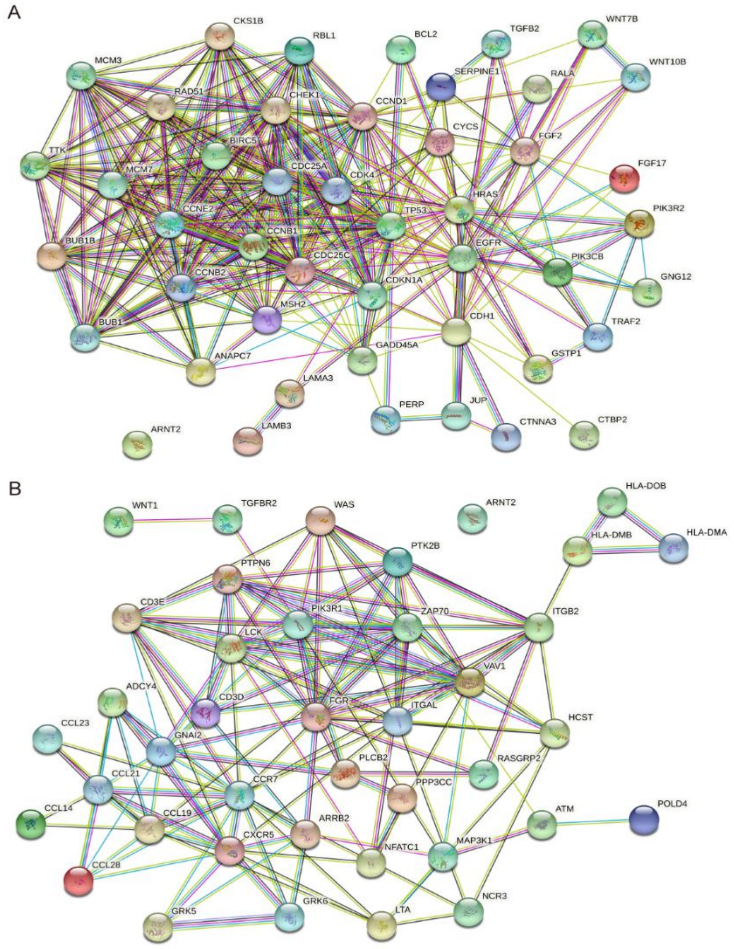

Supplementary Figure 3: PPI network ofARNT2-relatedtarget genes in NPC. (A)PPI network based on the genes of the first three KEGG pathways of intersection of up-regulated DEGs, ARNT2 positively correlated CEGs and target genes. (B) PPI network based on the genes of the first three KEGG pathways of intersection of down-regulated DEGs, ARNT2 negatively correlated CEGs and target genes.
